# Survival of patients on chronic hemodialysis versus chronic peritoneal dialysis

**DOI:** 10.17843/rpmesp.2022.392.10853

**Published:** 2022-06-30

**Authors:** Wilmer Guzman-Ventura, José Caballero-Alvarado

**Affiliations:** 1 Hospital Víctor Lazarte Echegaray, Seguro Social de Salud, Trujillo, Peru. Hospital Víctor Lazarte Echegaray Seguro Social de Salud Trujillo Peru; 2 Escuela de Medicina Humana, Universidad Privada Antenor Orrego, Trujillo, Peru. Universidad Privada Antenor Orrego Escuela de Medicina Humana Universidad Privada Antenor Orrego Trujillo Peru; 3 Hospital Regional Docente de Trujillo, Trujillo, Peru. Hospital Regional Docente de Trujillo Trujillo Peru; 4 Escuela de Posgrado, Universidad Privada Antenor Orrego, Trujillo, Peru. Universidad Privada Antenor Orrego Escuela de Posgrado Universidad Privada Antenor Orrego Trujillo Peru

**Keywords:** Survival, Survival Analysis, Dialysis, Mortality, Peritoneal dialysis

## Abstract

**Objective.:**

To compare the survival of patients with chronic kidney disease (CKD) on hemodialysis (HD) versus peritoneal dialysis (PD).

**Materials and methods.:**

Survival analysis of a retrospective cohort of patients ≥ 18 years who started HD versus PD at the Victor Lazarte Echegaray Hospital from 2015 to 2019. We analyzed the following covariates: age, sex, diabetes mellitus as cause of CKD, temporary central venous catheter (CVC) as initial vascular access and glomerular filtration rate. Survival was calculated with Kaplan-Meier curves for the overall cohort and for age ≥ 60 years, diabetes mellitus as a cause of CKD and CVC. The risk of death was estimated by Hazard Ratio (HR) according to the Cox proportional hazards model for each covariate adjusted for dialysis type in a bivariate and multivariate analysis considering significant difference if the p-value < 0.05.

**Results.:**

We included 368 patients on HD of whom 129 (35.1%) died, and 172 patients on PD of whom 66 (38.4%) died (p=0.455). The cumulative probability of survival at 60 months for HD was 30% and for PD was 37% with similar survival curves (p=0.719). The median survival time for HD was 32 months (IQR: 20-53) and for PD was 32.5 months (IQR: 18-57) (p=0.999). The covariates associated with higher mortality adjusted for dialysis type were age ≥60 years (HR 1.77; p<0.001) and diabetes mellitus as a cause of CKD (HR 1.63; p=0.002).

**Conclusions.:**

Survival of patients with CKD on HD and PD was similar.

## INTRODUCTION

Chronic kidney disease (CKD) is among the top 20 causes of disease burden globally ^1^. In 2019, ~1.5 million deaths were caused by CKD, three-quarters of which occurred in low- and middle-income countries (LMICs) and CKD is estimated to affect approximately 15% of the population aged 20 years or older ^1^ ^,^ ^2^; although national studies in Peru have reported higher prevalence ^3^. To reduce the impact of CKD, adequate clinical management of patients is necessary, especially in LMIC with a high prevalence of CKD such as Peru.

In patients with end-stage CKD, the mainstay of treatment is renal replacement therapy with dialysis ^4^, which prolongs life. In Peru there are two options for dialysis: hemodialysis (HD) and peritoneal dialysis (PD) ^5^. However, studies that have compared survival in HD and PD have not had uniform results; there are studies that show superiority in survival time using a specific type of dialysis (HD or PD) ^6^ ^,^ ^7^. A meta-analysis comparing mortality between HD and PD patients has reported similar survival times between both types, although with substantial heterogeneity among the included studies ^8^.

In La Libertad, one of the most populated departments of Peru, most patients with CKD requiring dialysis therapies are treated with both HD and PD, being the Victor Lazarte Echegaray Hospital (HVLE) the main care center. Considering the lack of global consensus on the best dialysis option, it is necessary and a priority to have local evidence on the survival time in HD versus PD, in addition to knowing what factors could be associated. Studies based on dialysis records can provide an idea of local survival in both dialysis modalities, as long as there is no evidence from clinical trials. Based on this background, the aim of this study was to compare the survival of patients with CKD treated at HVLE on HD versus PD and to relate it to covariates using the hospital’s medical records.

**KEY MESSAGES**

Motivation for the study: Chronic kidney disease is an important cause of mortality and requires renal replacement therapies such as hemodialysis and peritoneal dialysis, although it has not been determined which type is associated with better survival.

Main findings: Of 368 patients who started hemodialysis, 129 (35.1%) died and of 172 patients who started peritoneal dialysis, 66 (38.4%) died. Survival curves in hemodialysis and peritoneal dialysis were similar and were affected by age ≥ 60 years and diabetes *mellitus* as the cause of the disease.

Implications: The results suggest that the health care of diabetic patients and those aged ≥60 years should be maximized because they have a higher risk of mortality in both hemodialysis and peritoneal dialysis, with both types having equal survival.

## MATERIALS AND METHODS

### Design and type of research

Survival analysis of a retrospective cohort of patients with CKD who initiated HD and PD at HVLE between the years 2015 and 2019.

### Population and sample

The population consisted of all patients with CKD who started HD or PD at HVLE between 2015 and 2019. To identify the patients, we consulted the Archive and Medical Records Office and the HVLE Electronic Management System using the ICD-10 codes: N18.0, N18.6 and N18.9. This medical records registry has the patient database stored from 2015 through 2022. A sample size was not calculated because all patients who met the selection criteria were included. The unit of analysis was each patient with CKD who started HD or PD between 2015 and 2019.

All patients with CKD who started HD or PD between the years 2015 and 2019, of both sexes, over 18 years of age and who had a length of stay on HD or PD greater than or equal to three months were included. Patients with incomplete data records, patients who started HD or PD in another health center, patients with acute kidney damage or acute CKD, and patients who withdrew from the program due to recovery of renal function were excluded. In patients who for some reason changed from HD to PD or vice versa, only the first admission, whether HD or PD, was considered.

### Study variables

The exposure variable was the type of dialysis, the exposed population were patients who received HD and the unexposed population were patients who received PD. The outcome variables were overall mortality and overall survival time. The covariables were age, sex, diabetes mellitus as a cause of CKD, use of temporary central venous catheter as initial vascular access, and glomerular filtration rate.

From the medical records we extracted the following covariables for each individual: sex, age (years), dialysis type (HD or PD); diabetes *mellitus* as a cause of CKD (yes/no); use of temporary central venous catheter (CVC) as initial vascular access (yes/no); initial glomerular filtration rate (GFR) calculated with creatinine prior to dialysis admission; date dialysis was started and date of last control or death. The date of death was confirmed with the application of the National Computerized Death System (SINADEF).

For statistical analysis, age was categorized into two groups: ≥60 years and <60 years and GFR in mL/min/1.73 m^2^, estimated with the Modification of Diet in Renal Disease (MDRD) equation, was categorized into ≥10 mL/min/1.73 m^2^ and <10 mL/min/1.73 m^2^. The creatinine value used for this estimation was that closest to the time prior to the start of dialysis.

Using the dates of dialysis initiation and the date of last control or death, the survival of each patient was defined as the time (in months) from dialysis initiation (HD or PD) to the last control before December 31, 2019, or to the event of death ^9^ ^,^ ^10^.

The type of dialysis had two possible options: HD or PD. Hemodialysis therapy was defined as the regular program where the patient attended a hemodialysis center twice a day (15 of 368 patients, 4.0%) or three times a week (353 of 368 patients, 96.0%). PD therapy was defined as the regular program where the patient dialyzed at home more frequently than three times a day or automated peritoneal dialysis; were 82 (47.6%) patients in the PD program who initially entered hemodialysis through a temporary CVC and in less than three months moved to the peritoneal dialysis program and were therefore included in the PD cohort.

### Statistical analysis

Categorical variables were presented in absolute frequencies and percentages, quantitative variables in measures of central tendency and dispersion, including median and interquartile range for variables with non-normal distribution. To compare the distribution of patients on HD versus PD and of deceased and surviving patients, chi-square tests were used for categorical variables and the Mann Whitney U test for continuous variables, considering statistical significance if the value of p<0.05.

We carried out a bivariate analysis and calculated the Hazard Ratio and its 95% confidence intervals according to dialysis modality and covariates: age ≥ 60 years, diabetes *mellitus* as a cause of CKD, use of temporary CVC as initial vascular access and initial GFR ≥ 10 ml/min, considering statistical significance if the p-value < 0.05.

The multivariate analysis was conducted using the Cox regression model to evaluate the risk of death with Hazard Ratio and its 95% CI adjusted for dialysis type for the covariates: age ≥ 60 years, diabetes *mellitus* as a cause of CKD, use of temporary CVC as initial vascular access, and initial GFR ≥ 10 mL/min, considering statistical significance if the p-value < 0.05. Compliance with the proportional hazards assumptions was analyzed according to the likelihood ratio, verifying that the effect of each covariate is independent of each other and that the risks in each group are proportional.

Survival analysis was carried out using the Kaplan-Meier method, considering the probability of death as an event and survivors and patients who dropped out or were lost during the study period as censored. The results of this model are shown using survival curves. The probability of survival of the study cohort is described up to 60 months. The Long Rank statistical test was used to evaluate significant differences between survival curves, considering statistical significance if the p-value < 0.05. We used the SPSS statistical program (version 25) for the analyses and figures.

### Ethical considerations

This research was approved by the Graduate School and the Bioethics Committee of the Universidad Privada Antenor Orrego (UPAO) by Resolution 0339-2020 and was also approved by the Research and Ethics Committee of the Red Asistencial La Libertad de EsSalud (social health insurance) by means of “Constancia 69”. The data collected from the patients were treated respecting the principle of confidentiality. Informed consent was not requested because data were only reviewed and extracted from medical records without subjecting patients to any intervention.

## RESULTS

### Overall results

We analyzed 540 patients who initiated dialysis between the years 2015 to 2019 at HVLE. Of the total cohort, 368 (68.1%) patients started hemodialysis and 172 (31.8%) patients started peritoneal dialysis. 

Comparing the characteristics of patients who entered HD versus PD showed that the median age of patients on HD was 63 years (IQR: 54-72) versus 61 (IQR: 49-69) (p=0.016), had age ≥ 60 years 215 (58.4%) versus 87 (50.6%) (p=0.087), had diabetes *mellitus* as a cause of CKD 212 (57.6%) versus 77 (44.8%) (p=0.005) and presence of temporary CVC as initial vascular access in 235 (63.9%) versus 82 (47.7%) (p<0.001) (Table 1).

**Table 1:** General characteristics of hemodialysis and peritoneal dialysis patients.

General characteristics	Hemodialysis N = 368	Peritoneal dialysis N = 172	p-value
Deceased patients Surviving patients	129 (35.1%) 239 (64.9%)	66 (38.4%) 106 (61.6%)	0.455
Age, median (IQR)	63 (54-72)	61 (49-69)	0.016 ^a^
Age ≥ 60 years Age < 60 years	215 (58.4%) 153 (41.6%)	87 (50.6%) 85 (49.4%)	0.087
Male Female	199 (54.1%) 169 (45.9%)	88 (51.2%) 84 (48.8%)	0.489
Diabetes *mellitus* as a cause of CKD Other causes of CKD	212 (57.6%) 156 (42.4%)	77 (44.8%) 95 (55.2%)	0.005
CVC as initial vascular access Other type of access	235 (63.9%) 133 (36.1%)	82 (47.7%) 90 (52.3%)	<0.000
Initial GFR ≥ 10 mL/min/1.73 m^2^ Initial GFR < 10 mL/min/1.73 m^2^	18 (4.9%) 350 (95.1%)	12 (7.0%) 160 (63.0%)	0.324
Survival time, median (IQR: 25-75)	32 (20-53)	32.5 (18-57)	0.999^ a^

IQR: interquartile range; CKD: chronic kidney disease; CVC: central venous catheter; GFR: glomerular filtration rate.

a Mann-Whitney U test.

### Survival analysis

**Figure 1:**
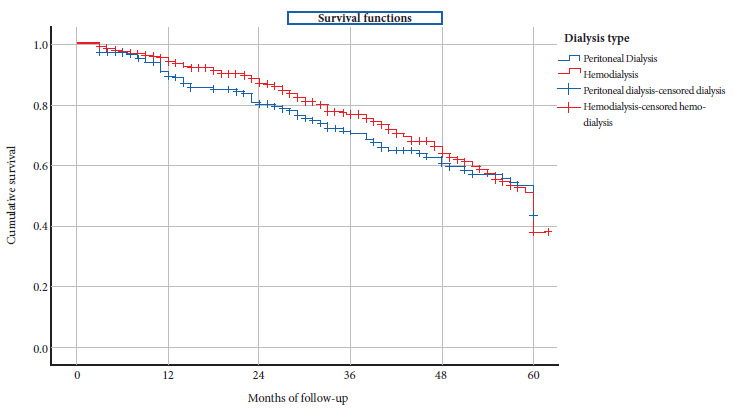
Survival curve of patients on hemodialysis and peritoneal dialysis.

The median survival time for HD versus PD patients was 32.5 months (IQR: 18-57) versus 32 (IQR: 20-53) (p = 0.999) (Table 1). The cumulative probability of survival at 60 months for HD versus PD was 30% versus 37% (p = 0.719) (Table 2) and had similar survival curves (Figure 1). The odds of survival in the study period for patients on HD versus PD were similar (HR: 1.095; 95% CI: 0.865-1.385; p = 0.455) (Table 2).

**Table 2:** Life table of hemodialysis and peritoneal dialysis patients.

Dialysis type	Starting month	Started	Withdrawn	Exposed	Deceased	Cumulative survival
Peritoneal dialysis	0	172	13	166	15	91%
	12	144	13	138	15	81%
	24	116	26	103	12	72%
	36	78	11	73	9	63%
	48	58	12	52	8	53%
	60	38	31	23	7	37%
Hemodialysis	0	368	34	351	16	95%
	12	317	40	298	22	88%
	24	256	62	225	29	77%
	36	165	32	149	21	66%
	48	112	19	103	23	51%
	60	70	52	44	18	30%

Of the total cohort of 540 patients, 195 (36.1%) died and 345 (73.9%) survived. Bivariate analysis adjusted for dialysis type showed that 136 (45.0%) patients ≥ 60 years died and 166 (55.0%) survived (HR: 2.067; 95% CI: 1.521-2.808; p < 0.001). Regarding patients with diabetes *mellitus* as a cause of CKD, 122 (42.2%) died and 167 (57.8%) survived (HR: 1.954; 95% CI: 1.452-2.630; p < 0.001) (Table 3).

**Table 3:** Bivariate analysis of survival of hemodialysis and peritoneal dialysis patients according to covariates.

Covariables	Deceased N = 195	Survivors N = 345	Hazard Ratio	95% CI	p-value
Hemodialysis patients	129 (35.1%)	239 (64.9%)	1.095	0.865 - 1.385	0.455
Patients on peritoneal dialysis	66 (38.4%)	106 (61.6%)			
Age ≥ 60 years	136 (45.0%)	166 (55.0%)	2.067	1.521 - 2.808	<0.001
Age < 60 years old	59 (24.8%)	179 (75.2%)			
Male	107 (37.3%)	180 (62.7%)	0.933	0.744 - 1.169	0.546
Female	88 (34.8%)	165 (65.2%)			
Diabetes *mellitus* as a cause of CKD	122 (42.2%)	167 (57.8%)	1.954	1.452 - 2.630	<0.001
Other causes of CKD	73 (29.1%)	178 (79.9%)			
CVC as initial vascular access	111 (35.0%)	206 (65.0%)	1.076	0.858 - 1.348	0.528
Other type of access	84 (37.7%)	139 (62.3%)			
Initial GFR ≥ 10 mL/min/1.73 m^2^	15 (50.0%)	11 (50.0%)	1.076	0.858 - 1.348	0.528
Initial GFR < 10 mL/min/1.73 m^2^	180 (35.3%)	330 (64.7%)	0.706	0.484 - 1.029	0.103

CKD, chronic kidney disease; CVC, central venous catheter; GFR, glomerular filtration rate; 95% CI, 95% confidence interval.

Cox multivariate analysis adjusted for dialysis type showed that the risk of death in patients ≥ 60 years had a HR: 1.77; (95% CI: 1.285-2.443; p < 0.001) and the risk of death in patients with diabetes mellitus as a cause of CKD had a HR: 1.63; (95% CI: 1.198-2.230; p < 0.002) (Table 4).

**Table 4:** Cox multivariate analysis of survival of crude hemodialysis and peritoneal dialysis patients and adjusted for dialysis type.

Covariables	Crude analysis			Adjusted analysis		
	HR	95% CI	p-value	HR	95% CI	p-value
Age ≥ 60 years	1.77	1.28-2.45	<0.000	1.77	1.28-2.44	< 0.001
Diabetes *mellitus* as a cause of CKD	1.70	1.24-2.32	0.001	1.63	1.19-2.23	0.002
Dialysis type	0.79	0.58-1.07	0.125	_	_	_
CVC as initial vascular access	1.31	0.98-1.75	0.067	_	_	_
Initial GFR ≥ 10 mL/min/1.73 m^2^	1.41	0.83-2.40	0.200	_	_	_
Sex	1.00	0.75-1.33	0.994	_	_	_

CKD: chronic kidney disease; CVC: central venous catheter; GFR: glomerular filtration rate.

**Figure 2:**
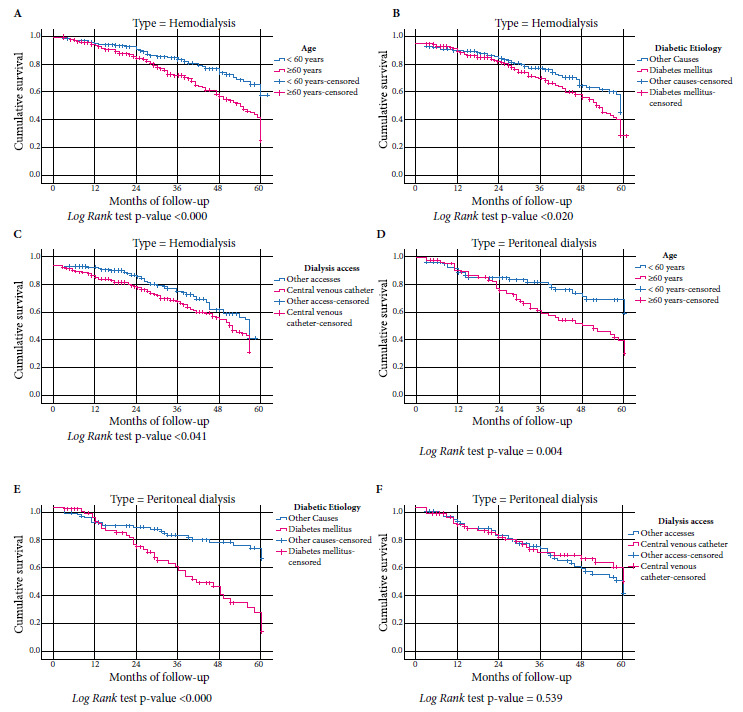
Survival curves of patients on hemodialysis according to age **(A)**; diabetic etiology **(B)**; start with temporary central venous catheter **(C)**. Survival curves of patients on peritoneal dialysis according to age **(D)**; diabetic etiology **(E)**; start with temporary central venous catheter **(F)**.

Survival curves stratified according to dialysis type showed that in hemodialysis and peritoneal dialysis patients age ≥ 60 years and those with diabetes *mellitus* as a cause of CKD have a higher risk of death (p < 0.05) (Figure 2) and only in hemodialysis patients the presence of temporary CVC as initial vascular access was associated with higher mortality (p = 0.041) (Figure 2).

## DISCUSSION

Using a five-year retrospective cohort design, we analyzed 540 patients with CKD who started dialysis (368 started HD and 172 started PD) in a referral center in La Libertad, Peru; these patients were followed for a minimum of three months and a maximum of 60 months. The cumulative probability of survival was similar for HD and PD. The mortality risk for HD and PD was higher in patients ≥ 60 years of age and in patients with diabetes *mellitus* as a cause of CKD, while the risk of mortality for HD was higher in patients who had temporary CVC as initial vascular access.

Several studies have compared survival curves in patients who initiate HD vs. PD, but they have obtained diverse and contradictory results. Rufino *et al*. ^7^ found better survival with PD during a three-year follow-up of 173 patients. On the other hand, Kim *et al*. ^11^, found similar survival time on HD vs. PD during a five-year follow-up of more than 30,000 patients. Similarly, Mehrotra *et al*. ^12^ found no difference in 64,406 patients who started PD and 620,020 HD patients. Likewise, Wong *et al*. ^13^ found no difference in survival at 7-year follow-up in an analysis of 1579 patients who started HD and 453 PD patients. Weinhandl *et al*. ^14^ analyzed a retrospective cohort of 6337 pairs of patients who started HD or PD in the United States and found better survival in patients who started peritoneal dialysis after five years of follow-up.

Those studies that found better survival in patients who initiate PD versus HD attributed it to improvements in technique, use of more biocompatible PD solutions, lower incidence of PD-related peritonitis episodes, and lower formation of advanced glycosylation end products ^7^ ^,^ ^15^ ^,^ ^16^. The differences in survival results reflect the conditions that each dialysis unit has to assign its patients and the conditions of each patient to best suit each type of therapy.

The results of our study represent the first local evidence that both types of dialysis have similar overall survival over a 5-year follow-up time. In HVLE dialysis patients, we find a cumulative probability of survival at 12, 24, 36, and 60 months on PD versus HD of 91% vs. 95%; 81% vs. 88%; 72% vs. 77%; and 37% vs. 30%, respectively. These results are similar to those found in other dialysis units in high-income countries ^6^ ^,^ ^17^.

In this study, patients ≥60 years who initiated HD and PD had a higher risk of mortality compared with patients <60 years. This finding is similar to that reported in other studies ^7^ ^,^ ^13^ ^,^ ^14^ ^,^ ^18^. The factors that explain the relationship between older age and higher mortality risk in dialysis patients are diverse. The older the patient, the greater the renal functional deterioration. Likewise, older patients generally have other comorbidities that, of course, imply a higher risk of mortality. Therefore, it is logical to consider that age is a major factor in the prognosis of patient survival.

This study shows that patients on dialysis with diabetes as the cause of their CKD had a higher risk of mortality; this finding is similar to international ^13^ ^,^ ^14^ and local studies ^19^ ^,^ ^20^. The presence of diabetic nephropathy as a risk factor for mortality in dialysis patients may be explained by a higher comorbidity index that adds disease burden and higher mortality to patients who start dialysis ^21^ ^,^ ^22^ ^,^ ^23^.

In this study, patients who initiated HD through a central venous catheter had a higher risk of death. Garcia *et al*. ^24^ evaluated the survival of 1110 patients who started HD or PD by arteriovenous fistula (AVF) or central venous catheter and found that the presence of a central venous catheter at the start of hemodialysis was associated with a HR of 2.270 (p < 0.001) and no significant differences when hemodialysis by AVF was compared with peritoneal dialysis. Similar results were found by Perl *et al*. ^25^ ^) ^in their study of 7412 patients who started peritoneal dialysis, 6663 hemodialysis patients with arteriovenous fistula and 24,437 patients who started hemodialysis by CVC (HR: 1.8 p = 0.001). The greatest mortality associated with the presence of a central venous catheter is infection, this was demonstrated by Coentrão *et al*. ^26^ in a study of 42 patients who started peritoneal dialysis, 59 patients who started hemodialysis by AVF and 42 patients who started hemodialysis by CVC, also by Gómez *et al*. ^27^ who analyzed the risk of early death in 557 patients who started hemodialysis in Lima and found a RR: 2.25 (95% CI 1.08-4.67).

In this study, it cannot be concluded that patients with glomerular filtration rate ≥ 10 mL/min/1.73 m^2^ who initiated peritoneal dialysis (7.0%) and hemodialysis (4.9%) have a higher or lower risk of death due to the small number of patients who initiated dialysis with such glomerular filtration rate. This aspect has been little explored in survival studies that compared patients who started peritoneal dialysis with patients who started hemodialysis; although most studies, especially on hemodialysis initiation, have not found a lower mortality when starting with a glomerular filtration rate ≥ 10 mL (min/1.73 m^2^) ^28^ ^-^ ^30^.

We found that patients who initiate HD versus PD at HVLE have similar survival during the 5-year follow-up. Although this finding is not based on a randomized controlled study, and does not favor the use of a specific dialysis type, it can serve as an input for clinical decision making in our center and centers similar to HVLE in Peru. Ideally, this finding has to be confirmed in controlled clinical studies, in other national dialysis units, with a larger number of patients and during a longer period of patient follow-up. Additionally, this study showed that mortality in HD and PD is higher in patients ≥ 60 years of age and in patients with diabetes *mellitus* as a cause of CKD, as well as in patients who have temporary CVC as initial vascular access in patients initiating HD which is consistent with the global evidence. Given this last finding, it would be reasonable to encourage the creation and strengthening of pre-dialysis programs that prioritize the creation of arteriovenous fistulas as vascular access for all patients starting hemodialysis.

This study compared the survival of patients with CKD treated with HD versus PD in the main referral center for dialysis in La Libertad, Peru. This study has the strength of having a relatively long follow-up period (five years) and analyzing all patients who started hemodialysis or PD. However, there are limitations that should be taken into account because they may introduce bias into the final results. First, the allocation of patients to peritoneal dialysis or hemodialysis was not randomized and did not have strict criteria for entering one modality or the other, and this led to significant differences in patients assigned to HD and PD in median age, patients with diabetes mellitus as a cause of CKD, and the presence of temporary CVC as initial vascular access; this limitation has been found in all the studies that have been cited, because it is difficult to perform randomized clinical trials due to the characteristics of the patients who are assigned to PD, such as better socioeconomic and cultural status that can ensure adequate compliance with the treatments. Second, being a retrospective study, the included variables depended on the clinical records and did not allow the inclusion of other covariates such as causes of death, dialysis quality, hemoglobin level, albumin level, nutritional status or degree of comorbidities that could have influenced the results. Third, there was a significant loss of patients during follow-up, mainly due to the change from HD to PD and vice versa, loss of insurance status by EsSalud and migration of patients to their places of origin because HVLE is a referral hospital; although it was not possible to determine the number of patients lost for each of these reasons because the data did not appear in the medical records. Consequently, the data collected did not allow us to explore the causes of death, causes of loss to follow-up, HD and PD quality characteristics, PD modalities (manual vs. automated), socioeconomic status, urgency and dialysis admission modality that other studies have shown to be associated with mortality in dialysis patients ^31^ ^,^ ^32^. Future studies could explore the effects of variables not included in this research.

In conclusion, the survival of patients with chronic kidney disease treated with hemodialysis versus peritoneal dialysis was similar. There was a higher risk of death in patients ≥ 60 years of age and with diabetes *mellitus* as a cause of chronic kidney disease.
