# Supplementary material for: Survival of patients on chronic hemodialysis versus chronic peritoneal dialysis
Source: Rev Peru Med Exp Salud Publica. 2022 Jun 30;39(2):161–9. doi: 10.17843/rpmesp.2022.392.10853 (PMC11397744; doi:10.17843/rpmesp.2022.392.10853)
Supplement: Translation

# Translation: Sobrevida de pacientes en hemodiálisis crónica versus diálisis peritoneal crónica

**Authors:** Wilmer Guzman-Ventura, José Caballero-Alvarado

**Affiliations:** 1 Hospital Víctor Lazarte Echegaray, Seguro Social de Salud, Trujillo, Perú.; 2 Escuela de Medicina Humana, Universidad Privada Antenor Orrego, Trujillo, Perú.; 3 Hospital Regional Docente de Trujillo, Trujillo, Perú.; 4 Escuela de Posgrado, Universidad Privada Antenor Orrego, Trujillo, Perú.

**Keywords:** Supervivencia, Análisis de Supervivencia, Diálisis, Mortalidad, Diálisis Peritoneal

## Abstract

**Objetivo.:**

Comparar la supervivencia de pacientes con enfermedad renal crónica (ERC) en hemodiálisis (HD) versus diálisis peritoneal (DP).

**Materiales y métodos.:**

Se realizó un análisis de supervivencia de una cohorte retrospectiva de pacientes ≥ 18 años que iniciaron HD versus DP en el Hospital Víctor Lazarte Echegaray del 2015 al 2019. Se analizaron las covariables: edad, sexo, diabetes *mellitus* con causa de ERC, catéter venoso central temporal (CVC) como acceso vascular inicial y tasa de filtración glomerular. Se calculó la supervivencia con curvas de Kaplan-Meier para la cohorte global y para edad ≥ 60 años, diabetes *mellitus* como causa de ERC y CVC. Se estimó el riesgo de fallecer mediante Hazard Ratio (HR) según el modelo de riesgos proporcionales de Cox para cada covariable ajustada a la modalidad de diálisis en un análisis bivariado y multivariado considerando diferencia significativa si el valor de p < 0,05.

**Resultados.:**

Se incluyeron 368 pacientes en HD de los cuales fallecieron 129 (35,1%) y 172 pacientes en DP de los cuales fallecieron 66 (38,4%) (p=0,455). La probabilidad acumulada de supervivencia a los 60 meses para HD fue de 30% y para DP fue de 37% con curvas de supervivencia similares (p=0,719). La mediana del tiempo de sobrevida en HD fue de 32 meses (RIC: 20-53) y en DP fue de 32,5 meses (RIC: 18-57) (p=0,999). Se determinó que las covariables asociadas con una mayor mortalidad ajustadas a la modalidad de diálisis fueron edad ≥60 años (HR 1,77; p<0,001) y diabetes *mellitus* como causa de ERC (HR 1,63; p=0,002).

**Conclusiones.:**

La supervivencia de los pacientes con enfermedad renal crónica en HD y DP fue similar.

## INTRODUCCIÓN

La enfermedad renal crónica (ERC) está entre las 20 primeras causas de carga de enfermedad a nivel global ^1^. En 2019, ~1,5 millones de muertes fueron causadas por la ERC, cuyas tres cuartas partes ocurrieron en países de mediano y bajo ingreso (LMIC, por sus siglas en inglés) y se estima que la ERC afecta aproximadamente al 15% de la población mayor o igual a 20 años.^1^ ^,^ ^2^; aunque estudios nacionales en Perú han reportado prevalencias mayores ^3^. Para reducir el impacto de la ERC es necesario un manejo clínico adecuado de los pacientes, especialmente en LMIC con alta prevalencia de ERC como el Perú.

En los pacientes con ERC en estado terminal, el pilar del tratamiento es la terapia de reemplazo renal con diálisis ^4^, que permite prolongar la vida. En el Perú existen dos opciones para hacer diálisis: hemodiálisis (HD) y diálisis peritoneal (DP) ^5^. Sin embargo, los estudios que han comparado la supervivencia en HD y DP no han tenido resultados uniformes; existen estudios que demuestran superioridad en el tiempo de supervivencia usando un tipo específico de diálisis (DH o DP) ^6^ ^,^ ^7^. Un metaanálisis que compara la mortalidad entre pacientes con HD y DP ha reportado tiempos similares de supervivencia entre ambos tipos, aunque con una heterogeneidad sustancial entre los estudios incluidos ^8^.

En La Libertad, uno de los departamentos más poblados del Perú, la mayoría de los pacientes con ERC que requieren terapias de diálisis son atendidos, tanto con HD como con DP, siendo el Hospital Víctor Lazarte Echegaray (HVLE) el principal centro de atención. Teniendo en cuenta la falta de consenso global sobre la mejor opción de diálisis, es necesario y prioritario tener evidencia local sobre el tiempo de supervivencia en HD versus DP, además de conocer qué factores podrían estar asociados. Los estudios en base a registros de diálisis pueden dar una idea de la supervivencia local en ambas modalidades de diálisis, mientras no se tenga evidencia de ensayos clínicos. Basados en estos antecedentes, el objetivo del estudio fue comparar la supervivencia de los pacientes con ERC atendidos en el HVLE en HD versus DP y relacionarlo con covariables usando los registros de historias clínicas del hospital.

**MENSAJE CLAVE**

Motivación para realizar el estudio: la enfermedad renal crónica es causa importante de mortalidad y requiere terapias de reemplazo renal como hemodiálisis y diálisis peritoneal, aunque no se ha determinado qué modalidad se asocia con mejor sobrevida.

Principales hallazgos: de 368 pacientes que iniciaron hemodiálisis fallecieron 129 (35,1%) y de 172 pacientes que iniciaron diálisis peritoneal fallecieron 66 (38,4%). Las curvas de supervivencia en hemodiálisis y diálisis peritoneal fueron similares y fueron afectadas por la edad ≥ 60 años y por la diabetes mellitus como causa de la enfermedad.

Implicancias: los resultados sugieren que se debe extremar los cuidados de salud de los pacientes diabéticos y en ≥60 años porque tienen mayor riesgo de mortalidad tanto en hemodiálisis como en diálisis peritoneal, teniendo ambas modalidades igual supervivencia.

## MATERIALES Y MÉTODOS

### Diseño y tipo de investigación

Análisis de supervivencia de una cohorte retrospectiva de pacientes con ERC que iniciaron HD y DP en el HVLE entre los años 2015 al 2019. 

### Población y muestra

La población estuvo conformada por la totalidad de pacientes con ERC que iniciaron HD o DP en el HVLE entre los años 2015 al 2019. Para identificar a los pacientes, se consultó en la Oficina de Archivo e Historias Clínicas y el Sistema de Gestión Electrónica del HVLE utilizando los códigos CIE-10: N18.0, N18.6 y N18.9. Este registro de historias clínicas tiene almacenado la base de datos de los pacientes desde el 2015 hasta el 2022. No se calculó un tamaño muestral porque fueron incluidos todos los pacientes que cumplieron con los criterios de selección. La unidad de análisis fue cada paciente con ERC que inició HD o DP entre 2015 al 2019.

Se incluyeron a todos los pacientes con ERC que iniciaron HD o DP entre los años 2015 al 2019, de ambos sexos, mayores de 18 años y que tuvieron un tiempo de permanencia en HD o DP mayor o igual a tres meses. Se excluyeron los pacientes que tenían registros de datos incompletos, pacientes que iniciaron HD o DP en otro centro de salud, pacientes con daño renal agudo o ERC agudizada, y pacientes que fueron retirados del programa por recuperación de función renal. En los pacientes que por alguna razón cambiaron de HD a DP o viceversa, solamente se consideró el primer ingreso, ya sea HD o DP.

### Variables de estudio

La variable de exposición fue la modalidad de diálisis, la población expuesta fueron los pacientes que recibieron HD y la población no expuesta los pacientes que recibieron DP. Las variables de desenlace fueron la mortalidad general y tiempo de sobrevida general. Las covariables fueron la edad, sexo, diabetes *mellitus* como causa de ERC, uso de catéter venoso central temporal como acceso vascular inicial y la tasa de filtración glomerular.

Por cada individuo se extrajeron las siguientes covariables a partir de las historias clínicas: sexo, edad (años), modalidad de diálisis (HD o DP); diabetes *mellitus* como causa de ERC (sí/no); uso de catéter venoso central (CVC) temporal como acceso vascular inicial (sí/no); tasa de filtración glomerular (TFG) inicial calculada con la creatinina previa al ingreso a diálisis; fecha de inicio de diálisis y la fecha de último control o del fallecimiento. La fecha de fallecimiento se confirmó con el aplicativo del Sistema Informático Nacional de Defunciones (SINADEF).

Para el análisis estadístico, la edad se categorizó en dos grupos: ≥60 años y <60 años y la TFG en mL/min/1,73 m^2^, estimada con la ecuación *Modification of Diet in Renal Disease* (MDRD) se categorizó en ≥10 mL/min/1,73 m^2^ y <10 mL/min/1,73 m^2^. El valor de la creatinina utilizada para esta estimación fue la más cercana al momento previo del inicio de la diálisis.

Usando las fechas de inicio de diálisis y la fecha de último control o fallecimiento, se definió la supervivencia de cada paciente como el tiempo (en meses) desde el inicio de diálisis (HD o DP) hasta el último control antes del 31 de diciembre del 2019 o hasta el evento de fallecimiento ^9^ ^,^ ^10^.

La modalidad de diálisis tuvo dos opciones posibles: HD o DP. La terapia con hemodiálisis se definió como aquel programa regular donde el paciente acudió a un centro de hemodiálisis con una frecuencia de dos veces (15 de 368 pacientes, 4,0%) o tres veces por semana (353 de 368 pacientes, 96,0%). La terapia con DP se definió como el programa regular donde el paciente realiza diálisis en su domicilio con una frecuencia mayor a tres veces al día o diálisis peritoneal automatizada, hubo 82 (47,6%) pacientes del programa de DP que inicialmente ingresaron a hemodiálisis a través de un CVC temporal y que en menos de tres meses pasaron al programa de diálisis peritoneal, por lo cual fueron incluidos en la cohorte de DP. 

### Análisis estadístico

Las variables categóricas se presentaron en frecuencias absolutas y en porcentajes, las variables cuantitativas en medidas de tendencia central y de dispersión, incluyendo la mediana y el rango intercuartílico para variables con distribución no normal. Para comparar la distribución de los pacientes en HD versus DP y de los pacientes fallecidos y sobrevivientes se usaron las pruebas de chi cuadrado para las variables categóricas y la prueba U de Mann Whitney para variables continuas, considerando significancia estadística si el valor de p<0,05.

Se efectuó un análisis bivariado y se calcularon *Hazard Ratio* y sus intervalos de confianza al 95% según la modalidad de diálisis y las covariables: edad ≥ 60 años, diabetes *mellitus* como causa de ERC, uso de CVC temporal como acceso vascular inicial y TFG inicial ≥ 10 ml/min, considerando significancia estadística si el valor p < 0,05.

Se realizó un análisis multivariado usando el modelo de regresión de Cox para evaluar el riesgo de fallecer con *Hazard Ratio* y su IC 95% ajustado a la modalidad de diálisis para las covariables: edad ≥ 60 años, diabetes *mellitus* como causa de ERC, uso de CVC temporal como acceso vascular inicial y TFG inicial ≥ 10 mL/min, considerando significancia estadística si el valor p < 0,05. Se analizó el cumplimiento de los supuestos de los riesgos proporcionales según la razón de verosimilitud, verificando que el efecto de cada covariable sea independiente uno del otro y que los riesgos en cada grupo sean proporcionales.

Se realizó el análisis de sobrevida con el método de Kaplan-Meier, considerando como evento la probabilidad de fallecer y como censuras los sobrevivientes y los pacientes que abandonaron o se perdieron en el periodo de estudio. Los resultados de este modelo se muestran usando curvas de supervivencia. La probabilidad de sobrevivir de la cohorte de estudio se describe hasta los 60 meses. Se utilizó la prueba estadística Long Rank para evaluar diferencias significativas entre las curvas de supervivencia, considerando significancia estadística si el valor de p < 0,05. Los análisis y figuras se realizaron en el programa estadístico SPSS (versión 25). 

### Consideraciones éticas

Esta investigación fue aprobada por la Escuela de Posgrado y el Comité de Bioética de la Universidad Privada Antenor Orrego (UPAO) mediante Resolución 0339-2020 y también fue aprobado por el Comité de Investigación y Ética de la Red Asistencial La Libertad de EsSalud (seguro social en salud) mediante Constancia 69. Los datos recolectados de los pacientes fueron tratados respetando el principio de confidencialidad. No se solicitó consentimiento informado porque solo se revisaron y extrajeron datos de historias clínicas sin someter a los pacientes a ninguna intervención. 

## RESULTADOS

### Resultados generales

Se analizaron 540 pacientes que iniciaron diálisis entre los años 2015 a 2019 en el HVLE. De la cohorte total, los pacientes que iniciaron hemodiálisis fueron 368 (68,1%) y los pacientes que iniciaron diálisis peritoneal fueron 172 (31,8%). Al comparar las características de los pacientes que ingresaron a HD versus DP se obtuvo que la mediana de la edad de los pacientes en HD fue 63 años (RIC: 54-72) versus 61 (RIC: 49-69) (p = 0,016), tuvieron una edad ≥ 60 años 215 (58,4%) versus 87 (50,6%) (p=0,087), tuvieron diabetes *mellitus* como causa de ERC 212 (57,6%) versus 77 (44,8%) (p = 0,005) y presencia de CVC temporal como acceso vascular inicial en 235 (63,9%) versus 82 (47,7%) (p < 0,001) (Tabla 1).

**Tabla 1:** Características generales de los pacientes de hemodiálisis y diálisis peritoneal

Características generales	Hemodiálisis N = 368	Diálisis peritoneal N = 172	Valor de p
Pacientes fallecidos	129 (35,1%)	66 (38,4%)	0,455
Pacientes sobrevivientes	239 (64,9%)	106 (61,6%)	
Edad, mediana (RIC)	63 (54-72)	61 (49-69)	0,016 ^a^
Edad ≥ 60 años	215 (58,4%)	87 (50,6%)	0,087
Edad < 60 años	153 (41,6%)	85 (49,4%)	
Masculinos	199 (54,1%)	88 (51,2%)	0,489
Femeninos	169 (45,9%)	84 (48,8%)	
Diabetes mellitus como causa de ERC	212 (57,6%)	77 (44,8%)	0,005
Otras causas de ERC	156 (42,4%)	95 (55,2%)	
CVC como acceso vascular inicial	235 (63,9%)	82 (47,7%)	<0,000
Otro tipo de acceso	133 (36,1%)	90 (52,3%)	
TFG inicial ≥ 10 mL/min/1,73 m^2^	18 (4,9%)	12 (7,0%)	0,324
TFG inicial < 10 mL/min/1,73 m^2^	350 (95,1%)	160 (63,0%)	
Tiempo de supervivencia, mediana (RIC)	32 (20-53)	32,5 (18-57)	0,999^ a^

RIC: rango intercuartílico, ERC: enfermedad renal crónica, CVC: catéter venoso central, TFG: tasa de filtración glomerular.

a Prueba U de Mann-Whitney.

### Análisis de supervivencia

La mediana del tiempo de supervivencia para los pacientes de HD versus DP fue 32,5 meses (RIC: 18-57) versus 32 (RIC: 20-53) (p = 0,999) (Tabla 1). La probabilidad acumulada de supervivencia a los 60 meses en HD versus DP fue 30% versus 37% (p = 0,719) (Tabla 2) y fueron similares las curvas de supervivencia (Figura 1). Las probabilidades de sobrevivir en el periodo de estudio para los pacientes en HD versus DP fueron similares (HR: 1,095; IC 95%: 0,865-1,385; p = 0,455) (Tabla 2).

**Figura 1:** Curva de supervivencia de los pacientes en hemodiálisis y diálisis peritoneal

**Tabla 2:** Tabla de vida de los pacientes de hemodiálisis y diálisis peritoneal

Modalidad de diálisis	Mes de inicio	Ingresan	Retirados	Expuestos	Fallecidos	Supervivencia acumulada
Diálisis peritoneal	0	172	13	166	15	91%
	12	144	13	138	15	81%
	24	116	26	103	12	72%
	36	78	11	73	9	63%
	48	58	12	52	8	53%
	60	38	31	23	7	37%
Hemodiálisis	0	368	34	351	16	95%
	12	317	40	298	22	88%
	24	256	62	225	29	77%
	36	165	32	149	21	66%
	48	112	19	103	23	51%
	60	70	52	44	18	30%

De la cohorte total de 540 pacientes, fallecieron 195 (36,1%) y sobrevivieron 345 (73,9%). El análisis bivariado ajustado a la modalidad de diálisis demostró que en pacientes ≥ 60 años fallecieron 136 (45,0%) y sobrevivieron 166 (55,0%) (HR: 2,067; IC 95%: 1,521-2,808; p < 0,001), en pacientes con diabetes *mellitus* como causa de ERC fallecieron 122 (42,2%) y sobrevivieron 167 (57,8%) (HR: 1,954; IC 95%: 1,452-2,630; p < 0,001) (Tabla 3).

**Tabla 3:** Análisis bivariado de sobrevida de pacientes de hemodiálisis y diálisis peritoneal según covariables

Covariables	Fallecidos N = 195	Sobrevivientes N = 345	Hazard Ratio	IC 95%	Valor de p
Pacientes en hemodiálisis	129 (35,1%)	239 (64,9%)	1,095	0,865 - 1,385	0,455
Pacientes en diálisis peritoneal	66 (38,4%)	106 (61,6%)			
Edad ≥ 60 años	136 (45,0%)	166 (55,0%)	2,067	1,521 - 2,808	<0,001
Edad < 60 años	59 (24,8%)	179 (75,2%)			
Masculinos	107 (37,3%)	180 (62,7%)	0,933	0,744 - 1,169	0,546
Femeninos	88 (34,8%)	165 (65,2%)			
Diabetes* mellitus *como causa de ERC	122 (42,2%)	167 (57,8%)	1,954	1,452 - 2,630	<0,001
Otras causas de ERC	73 (29,1%)	178 (79,9%)			
CVC como acceso vascular inicial	111 (35,0%)	206 (65,0%)	1,076	0,858 - 1,348	0,528
Otro tipo de acceso	84 (37,7%)	139 (62,3%)			
TFG inicial ≥ 10 mL/min/1,73 m^2^	15 (50,0%)	11 (50,0%)	1,076	0,858 - 1,348	0,528
TFG inicial < 10 mL/min/1,73 m^2^	180 (35,3%)	330 (64,7%)	0,706	0,484 - 1,029	0,103

ERC: enfermedad renal crónica, CVC: catéter venoso central, TFG: tasa de filtración glomerular, IC 95%: intervalo de confianza al 95%.

El análisis multivariado de Cox ajustado a la modalidad de diálisis demostró que el riesgo de fallecer en pacientes ≥ 60 años tuvo un HR: 1,77; IC 95%: 1,285-2,443; p < 0,001) y el riesgo de fallecer en pacientes con diabetes *mellitus* como causa de ERC tuvo un HR: 1,63; IC 95%: 1,198-2,230; p < 0,002) (Tabla 4).

**Tabla 4:** Análisis multivariado de Cox de sobrevida de pacientes de hemodiálisis y diálisis peritoneal crudos y ajustado a la modalidad de diálisis

Covariables	Análisis crudo			Análisis ajustado		
	HR	IC 95%	Valor de p	HR	IC 95%	Valor de p
Edad ≥ 60 años	1,77	1,28-2,45	<0,000	1,77	1,28-2,44	< 0,001
Diabetes mellitus como causa de ERC	1,70	1,24-2,32	0,001	1,63	1,19-2,23	0,002
Modalidad de diálisis	0,79	0,58-1,07	0,125	_	_	_
CVC como acceso vascular inicial	1,31	0,98-1,75	0,067	_	_	_
TFG inicial ≥ 10 mL/min/1,73 m^2^	1,41	0,83-2,40	0,200	_	_	_
Sexo	1,00	0,75-1,33	0,994	_	_	_

ERC: enfermedad renal crónica, CVC: catéter venoso central, TFG: tasa de filtración glomerular

Las curvas de supervivencia estratificadas según la modalidad de diálisis demostraron que en pacientes de hemodiálisis y diálisis peritoneal la edad ≥ 60 años y la diabetes *mellitus* como causa de ERC tienen mayor riesgo de fallecer (p < 0,05) (Figura 2) y solo en pacientes de hemodiálisis la presencia de CVC temporal como acceso vascular inicial se asocia con mayor mortalidad (p = 0,041) (Figura 2).

**Figura 2:** Curvas de supervivencia de pacientes en hemodiálisis según edad **(A)**; etiología diabética **(B)**; inicio por catéter venoso central temporal **(C)**. Curvas de supervivencia de pacientes en diálisis peritoneal según edad** (D)**; etiología diabética** (E)**; inicio por catéter venoso central temporal **(F)**.

## DISCUSIÓN

Usando un diseño de cohorte retrospectiva de cinco años de seguimiento se analizaron 540 pacientes con ERC que iniciaron diálisis (368 iniciaron HD y 172 iniciaron DP) en un centro de referencia de la Libertad, Perú; estos pacientes fueron seguidos por un periodo mínimo de tres meses y máximo de 60 meses. La probabilidad acumulada de supervivencia fue similar en HD y DP. El riesgo de mortalidad en HD y DP fue mayor en los pacientes ≥ de 60 años y en los pacientes con diabetes *mellitus* como causa de ERC, en tanto que el riesgo de mortalidad en HD fue mayor en los pacientes que tuvieron CVC temporal como acceso vascular inicial. 

Los diversos estudios que han comparado las curvas de supervivencia en pacientes que inician HD vs. DP han tenido resultados diversos y contradictorios. Rufino *et al.* ^7^ ^) ^encontraron una mejor supervivencia con DP durante un seguimiento de tres años de 173 pacientes. Por otro lado, Kim *et al.* ^11^, encontraron un tiempo similar de supervivencia en HD vs. DP durante un seguimiento de cinco años de más de 30 000 pacientes. De forma similar, Mehrotra *et al*. ^12^, no encontraron diferencias en 64 406 pacientes que iniciaron DP y 620 020 de HD. Asimismo, Wong *et al.* ^13^ no encontraron diferencias en la supervivencia a siete años de seguimiento en un análisis de 1579 pacientes que iniciaron HD y 453 pacientes de DP. Weinhandl *et al.* ^14^ ^) ^analizaron una cohorte retrospectiva de 6337 pares de pacientes que iniciaron HD o DP en Estados Unidos y encontraron una mejor supervivencia en los pacientes que iniciaron diálisis peritoneal en cinco años de seguimiento. 

Aquellos estudios que encontraron una mejor supervivencia en pacientes que inician DP versus HD lo atribuyeron a mejoras en la técnica, uso de soluciones de DP más biocompatibles, menor incidencia de episodios de peritonitis relacionada a la DP, y una menor formación de productos finales de la glicosilación avanzada^ (^ ^7^ ^,^ ^15^ ^,^ ^16^. Las diferencias en los resultados de supervivencia reflejan las condiciones que tiene cada unidad de diálisis para asignar sus pacientes y las condiciones de cada paciente para adecuarse mejor a cada tipo de terapia. 

Los resultados del presente estudio representan la primera evidencia local de que ambos tipos de diálisis tienen una supervivencia general similar durante un tiempo de seguimiento de cinco años. En los pacientes con diálisis del HVLE, se encuentra una probabilidad acumulada de supervivencia a los 12, 24, 36 y 60 meses en DP versus HD de 91% vs. 95%; 81% vs. 88%; 72% vs. 77% y 37% vs. 30%, respectivamente. Estos resultados son similares a lo encontrado en otras unidades de diálisis de países de altos ingresos ^6^ ^,^ ^17^.

En el presente estudio, los pacientes ≥60 años que iniciaron HD y DP tuvieron mayor riesgo de mortalidad comparado con los pacientes <60 años. Este hallazgo es similar a lo reportado en otros estudios ^7^ ^,^ ^13^ ^,^ ^14^ ^,^ ^18^. Los factores que explican la relación entre una mayor edad y un mayor riesgo de mortalidad en pacientes en diálisis son diversos. A mayor edad existe mayor deterioro funcional renal. Asimismo, los pacientes con mayor edad generalmente tienen otras comorbilidades que, desde ya, implican un mayor riesgo de mortalidad. Por lo tanto, resulta lógico considerar que la edad sea un factor primordial en el pronóstico de la supervivencia de los pacientes. 

Este estudio demuestra que los pacientes en diálisis cuya causa de su ERC fue diabetes tuvieron un mayor riesgo de mortalidad; este hallazgo es similar a estudios internacionales ^13^ ^,^ ^14^ y locales ^19^ ^,^ ^20^. La presencia de nefropatía diabética como factor de riesgo de mortalidad en los pacientes en diálisis puede explicarse por un mayor índice de comorbilidad que agrega carga de enfermedad y mayor mortalidad a los pacientes que inician diálisis ^21^ ^,^ ^22^ ^,^ ^23^.

En el presente estudio, los pacientes que iniciaron HD por un catéter venoso central tuvieron mayor riesgo de muerte. García *et al.* ^24^ ^) ^evaluaron la supervivencia de 1110 pacientes que iniciaron HD o DP por fístula arteriovenosa (FAV) o por catéter venoso central y encontraron que la presencia de catéter venoso central al inicio de la hemodiálisis se asociaba con un HR de 2,270 (p < 0,001) y sin diferencias significativas cuando se comparaba la hemodiálisis por FAV con diálisis peritoneal. Similares resultados encuentran Perl *et al.* ^25^ en su estudio de 7412 pacientes que iniciaron diálisis peritoneal, 6663 pacientes de hemodiálisis con fístula arteriovenosa y 24 437 pacientes que iniciaron hemodiálisis por CVC (HR: 1,8 p = 0,001). La mayor mortalidad asociada con la presencia de un catéter venoso central es la infección, esto fue demostrado por Coentrão *et al*.^ (^ ^26^ ^) ^en un estudio de 42 pacientes que iniciaron diálisis peritoneal, 59 pacientes que iniciaron hemodiálisis por FAV y 42 pacientes que iniciaron hemodiálisis por CVC, también por Gómez *et al.* ^(^ ^27^ que analizaron el riesgo de muerte precoz en 557 pacientes que iniciaron hemodiálisis en Lima y encontraron un RR: 2,25 (IC 95% 1,08-4,67). 

En este estudio, entre los pacientes con tasa de filtración glomerular ≥ 10 mL/min/1,73 m^2 ^que iniciaron diálisis peritoneal (7,0%) y hemodiálisis (4,9%) no se puede concluir que tengan mayor o menor riesgo de muerte debido a la poca cantidad de pacientes que iniciaron diálisis con dicha tasa de filtración glomerular. Este aspecto ha sido poco explorado en los estudios de supervivencia que compararon pacientes que iniciaron diálisis peritoneal con pacientes que iniciaron hemodiálisis; aunque la mayoría de los estudios, sobre todo de inicio de hemodiálisis, no ha encontrado una menor mortalidad al iniciar con tasa de filtración glomerular ≥ 10 mL (min/1,73 m^2 (^ ^28^ ^-^ ^30^. 

Se encontró que los pacientes que inician HD versus DP atendidos en el HVLE tienen una supervivencia similar durante los cinco años de seguimiento. Si bien este hallazgo no está basado en un estudio controlado y aleatorizado, y no favorece el uso de una modalidad específica de diálisis, puede servir como insumo en la toma de decisiones clínicas en nuestro centro y centros similares al HVLE de Perú. Idealmente, este hallazgo tiene que ser confirmado en estudios clínicos controlados, en otras unidades de diálisis nacionales, con un mayor número de pacientes y durante un mayor periodo de seguimiento de pacientes. Adicionalmente, este estudio demostró que la mortalidad en HD y DP es mayor en pacientes ≥ de 60 años y en pacientes con diabetes *mellitus* como causa de ERC, así como en pacientes que tienen CVC temporal como acceso vascular inicial en pacientes que inician HD lo cual es consistente con la evidencia global. Considerando este último hallazgo, resultaría razonable incentivar la creación y fortalecimiento de programas prediálisis en donde se priorice la creación de fístulas arteriovenosas como acceso vascular para todos los pacientes que inicien hemodiálisis.

En este estudio se comparó la supervivencia de pacientes con ERC atendidos con HD versus DP en el principal centro de referencia para diálisis de La Libertad, Perú. Este trabajo posee la fortaleza de tener un periodo de seguimiento relativamente largo (cinco años) y analizar a todos los pacientes que iniciaron hemodiálisis o DP. Sin embargo, hay limitaciones que se deben tener en cuenta porque pueden incorporar sesgos a los resultados definitivos. En primer lugar, la asignación de pacientes a diálisis peritoneal o hemodiálisis no fue aleatoria, no tuvo criterios estrictos para ingresar a una modalidad u otra, y esto generó que existan diferencias significativas en los pacientes asignados a HD y DP en la mediana de la edad, pacientes con diabetes *mellitus* como causa de ERC y presencia de CVC temporal como acceso vascular inicial; esta limitación se ha encontrado en todos los estudios que se han referenciado, porque es difícil realizar ensayos clínicos aleatorizados por las características de los pacientes que se asignan a DP, tales como mejor estado socioeconómico y cultural que pueda asegurar un cumplimiento adecuado de los tratamientos. En segundo lugar, al ser un estudio retrospectivo, las variables incluidas dependieron de las historias clínicas recolectadas y no permitieron que se incluyan otras covariables como causas de fallecimiento, calidad de diálisis, nivel de hemoglobina, nivel de albúmina, estado nutricional o grado de comorbilidades que pudieron influir en los resultados. En tercer lugar, hubo una pérdida importante de pacientes en el seguimiento, debido principalmente al cambio de modalidad de HD a DP y viceversa, pérdida de la condición de asegurado por EsSalud y migración de los pacientes a sus lugares de origen por ser el HVLE un hospital de referencia; aunque no se pudo determinar la cantidad de pacientes perdidos por cada una de estas razones por no figurar los datos en las historias clínicas. En consecuencia, con los datos recolectados no se llegó a explorar las causas de muerte, causas de la pérdida de seguimiento, características de la calidad de la HD y DP, modalidades de la DP (manual vs. automatizada), estado socioeconómico, urgencia y modalidad de ingreso a diálisis que otros estudios han demostrado que se asocian con mortalidad de pacientes en diálisis ^31^ ^,^ ^32^. Futuros estudios podrían explorar los efectos de las variables no registradas en este trabajo.

En conclusión, la supervivencia de los pacientes con enfermedad renal crónica atendidos con hemodiálisis versus diálisis peritoneal fue similar. Existió un mayor riesgo de fallecer en los pacientes ≥ 60 años y con diabetes *mellitus* como causa de la enfermedad renal crónica.
